# 3D Food Printing: Principles of Obtaining Digitally-Designed Nourishment

**DOI:** 10.3390/nu13103617

**Published:** 2021-10-15

**Authors:** Rodica-Anita Varvara, Katalin Szabo, Dan Cristian Vodnar

**Affiliations:** 1 Faculty of Food Science and Technology, University of Agricultural Sciences and Veterinary Medicine, Calea Mănăștur 3-5, 400372 Cluj-Napoca, Romania; varvara.anita@yahoo.com (R.-A.V.); katalin.szabo@usamvcluj.ro (K.S.); 2 Institute of Life Sciences, University of Agricultural Sciences and Veterinary Medicine, Calea Mănăștur 3-5, 400372 Cluj-Napoca, Romania

**Keywords:** 3D food printing, binding agents, by-products, coloring agents, fortifying constituents, personalized nutrition

## Abstract

Three-dimensional printing (3DP) technology gained significance in the fields of medicine, engineering, the food industry, and molecular gastronomy. 3D food printing (3DFP) has the main objective of tailored food manufacturing, both in terms of sensory properties and nutritional content. Additionally, global challenges like food-waste reduction could be addressed through this technology by improving process parameters and by sustainable use of ingredients, including the incorporation of recovered nutrients from agro-industrial by-products in printed nourishment. The aim of the present review is to highlight the implementation of 3DFP in personalized nutrition, considering the technology applied, the texture and structure of the final product, and the integrated constituents like binding/coloring agents and fortifying ingredients, in order to reach general acceptance of the consumer. Personalized 3DFP refers to special dietary necessities and can be promising to prevent different non-communicable diseases through improved functional food products, containing bioactive compounds like proteins, antioxidants, phytonutrients, and/or probiotics.

## 1. Introduction

Three-dimensional printing (3DP), also known by the terms “Additive Manufacturing” (AM) or/and “Rapid Prototyping”, is the process of making three-dimensional solid objects from a digital file fort [1]. This recent technology is acquiring a wide interest from researchers and various industries worldwide, because it is a versatile option for manufacturing and it offers new perspectives of expansion [1,2]. AM is based on a digitally-controlled robotic construction process, which is able to build up complex, solid forms layer by layer, and utilizes chemical reactions or phase transitions for binding the layers together [3,4]. With further development on the success of this technology, there is real potential for 3D printers to evolve through mass production and reach private homes. Heterogeneity of the material, and standardization of the protocols are important as well. In the last 10 years, several areas like medical fields, technical engineering, the army, and others adopted 3DP as an innovative approach [5].

The advancement of 3DP technology has gained importance in the fields of the food industry as well as in gastronomy. Researchers and inventors revealed its revolutionary potential through the advantages of functionality, increased flexibility in design, and of removing the risk of human inaccuracy in production [6]. The engineering concept of 3DP is based on a controlled robotic process with dedicated software (generally a Computer-Aided Design) that builds up the product, allowing designers to create a prototype in a short time, with a specific format (e.g., STL) [7]. 3D printers can use a variety of materials to form complex shapes depending on the rheological properties of the materials, like flow behavior, viscosity, and/or shear rate. Materials like metal, polymer filaments, and, lately, edible ingredients such as sugar, chocolate, and doughs, can be appropriate for 3D printing techniques, by adjusting flow properties and homogenous composition, in order to maintain their structure during and after the printing process [1].

### Background

The first appearance of 3D printers was back in 1983, when an American inventor, developed the technology of “stereolithography” which was used to create replacement components in industry, however, the 3D printers/3D products managed to find their way into art and private homes [8]. When it comes to the food sector, worldwide applications of 3DFP can be related to chocolate figures, cookie dough, and cheese profiles, elaborated by a group of students, in 2006, when the first multi-material 3D printer was used to obtain different food products [9]. Between 2006 and 2009, large sugar sculptures were printed, by using a hot-air beam to selectively melt and fuse sugar grains together [10]. Another printer, equipped with a refillable syringe-like dual extruder, was used to print any liquid material or paste type, like chocolate, peanut butter, ice-cream, marshmallow, jam, honey, ketchup, mustard, cream cheese, and cheese [9]. Other printers were used to create sculptures from cookie batter, mashed potatoes, crushed nuts, or thin-layer foodstuffs, like pancakes [9,11].

3DFP has as its main purpose tailored manufacturing of food in terms of sensory properties and nutritional content, at the same time [12]. Additionally, in the context of the circular economy strategies, food waste could be decreased through the use of agro-industrial by-products in printed nourishment [5,13,14].

Several technologies can be used in 3DP, nevertheless, the most commonly used in 2020 are Fused Deposition Modelling (FDM), known as material extrusion, followed by Selective Laser Sintering (SLS), due to the large variety of materials that can be printed. In the food industry, four of the 3DP techniques have been used so far, such as material extrusion, binder jetting, selective laser sintering, and inkjet printing, techniques which will be extensively discussed in the followings [2].

3DP has the potential to considerably modify business models, impact the global economy and change existing supply chains. Regarding the economical perspective, the value of the entire 3DP market was estimated to 6422.5 million USD in 2019 and it is expected to grow to 44,520 million USD by 2026, with a compound annual growth rate (CAGR) of 31.4% between 2021 and 2026 [9]. The key factors driving the growth of this market are the facile development of customized products, the reduction in manufacturing costs and process downtime, together with the development of new industrial-grade 3DP materials. However, limitation of product size, deficiency of standard process control, partial availability, and the high fee of materials are the factors that might limit its evolution, per general. The global 3D food printing (3DFP) market was estimated in 2016 at 8.75 million USD and it is expected to reach the value of 400 million USD, in the interval of 2017–2024, with a CAGR growth of 50%. North America and Europe are qualifying as the most important market leaders due to the technology democratization and its application among the food-service channels [9].

The aim of the present review is to highlight the advantages of 3DFP implementation in personalized or targeted nutrition, for example, the diet of the elderly could be supplemented with active ingredients to prevent specific disorders. Sensorial proprieties, texture, and structure of 3D-printed food play an important role in the acceptance of 3D food products, therefore, the main technologies applied and possible enhancements of quality properties through incorporation of fortifying constituents into the final food product will be discussed. Furthermore, as an alternative source of bioactive compounds, the reintegration of agro-industrial by-products into new food formulations will be approached in order to find sustainable solutions for both personalized nourishment and food-waste reduction.

## 2. Technology of 3DFP

3DFP is the development of food products using the techniques of AM. The 3D printers have pre-loaded recipes in their software, which allows the user to remotely design the products on their smart devices (e.g., phones, computers). Most frequently, the printing material is held by the syringes and is stratified layer by layer through a nozzle to obtain the shaped final food product. 

This mechanism provides the possibility of integrating bioactive components (e.g., carotenoids, anthocyanins) into foodstuffs to obtain a suitable diet for people with specific nutritional needs [5,15,16]. Various meals can be obtained in different shapes and colors, as shown in Table 1. For instance, Uribe-Wandurraga et al., (2020) obtained a cookie dough with various concentrations of microalgae *Arthrospira platensis* and *Chlorella vulgaris;* the shades of green were dose-dependently exposed, and the variation of color made the cookies more palatable and desirable [17].

The potential of standardized texture of the food is another benefit given by 3DFP technology, due to the possibility of obtaining identical texture constantly, compared to the texture-modified products made by hand [5]. Further, the products obtained through 3DFP technology can be adjustable for people with special diets, like pregnant women, astronauts or vegan/vegetarians [2,36]. Another advantage of 3DFP technology is related to the development of a variety of functional food products, by integrating diverse ingredients. For instance, Zhenbin Liu et al., (2020) integrated probiotics (*Bifidobacterium animalis* subsp. *lactis)* into 3D-printed mashed potatoes. The formulations were optimized and their 3DFP performance was evaluated and correlated with the rheological properties, and the feasibility studies of integrating the probiotics into the formulations. The results showed that during 12-day storage interval at 5°C the viability of the microorganisms was not significantly affected, indicating the possibility of food fortification with probiotics [24,37,38].

**Figure 1:**
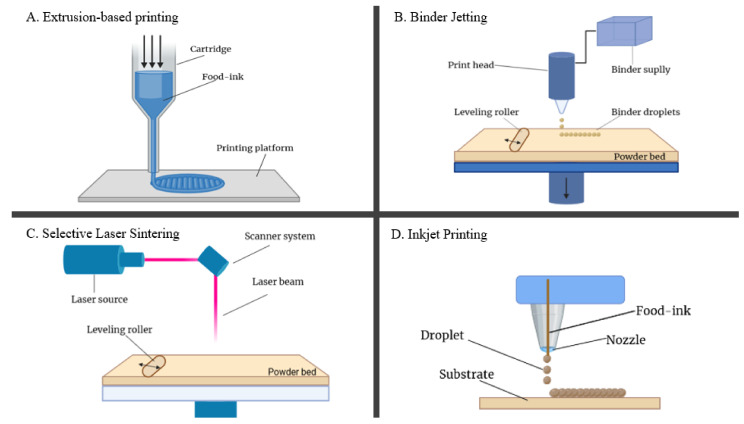
Concept figures for 3DFP technologies.

As a technical observation, to obtain the 3D-printed figure, there are three major factors that impact the final product’s quality, such as the printing material’s properties (e.g., viscosity, mesh size of the powder), process parameters (e.g., nozzle diameter, printing speed) and post-processing treatments (e.g., baking, boiling, microwaving, and frying) [11]. According to recent studies, the most relevant methods used in 3DFP are extrusion method, binder jetting, selective laser sintering, and inkjet printing. These methods are presented in Figure 1 and they will be described in the following [1,2,11]. 

### 2.1. Extrusion Technology of 3DFP

The concept of extrusion technology is based on soft materials, using a temperature control or semi-solid viscous system [2]. Material is held in a cartridge, drawn through a nozzle, where it can be heated, and is deposited layer by layer on the printer’s platform through the horizontal movements of the nozzle and the vertical movement of the platform. In hot-melt extrusion (HME), high temperature is applied to the material via a syringe or a heating block. An optimal temperature of the material is maintained to control its viscosity and to make it flowable through the nozzle [2]. HME has various applications in healthcare (medical devices and mixed active pharmaceutical ingredients) and the plastics industry, and it also has been applied in food extrusion for different materials, like pre-tempered chocolate, food and by-products puree, meat puree, or even cheese and doughs [2,11]. The method has been used to print potato starch gel figures at different temperatures and the samples presented optimal printability at the concentration of 15–25% starch suspension at 70 °C [28].

The extrusion process has many factors that influence the quality of the final product and has great potential and viability when these factors are handled successfully. The mechanical and the rheological properties of the material can influence the bonding of material layers through temperature control or by using chemical agents and/or food additives [2,28]. Feasible elements for the extrusion method are fresh food ingredients, like fruits and vegetables, as they can be blended and liquefied. However, according to Mantihal et al. (2020), the fresh food smoothies cannot sustain themselves in a well-defined shape after extrusion, therefore, food additives, like hydrocolloids (e.g., starch, gelatin xanthan gum, guar gum, pectin, etc.) are necessary to facilitate printability, flowability, and solidification [2]. Some other examples of usable materials in the extrusion method can be meat or seafood products, though it is indicated to maintain the temperature below 4 °C throughout all phases of the process in order to prevent microbial growth and to avoid food contamination. In this case, the 3D printer needs a cooling system attached [39]. Furthermore, other 3D structures were obtained from cookie dough [20], pectin-based formulations [10], and even gels based on xanthan/konjac gums [6].

A notable factor influencing the 3D-printed food’s quality is represented by the pressure in the nozzle during the printing process. To get precise results, this parameter has to be kept stable and at a constant level while printing [11]. Typical range pressure for printing soft foods (e.g., fish, beet puree, egg white foam, etc.) is between 20 and 50 kPa [40], for stiffer pastes (e.g., Vegemite, Marmite, etc.) is between 100 and 170 kPa [41], and for the third category, pastes with thicker consistency (multicomponent food matrices, rich in protein, fibers, etc., or with low water content) the pressure range is between 300–600 kPa [42,43,44].

In general, to develop fine resolution 3D items, the nozzle’s diameter is of vital importance, and it should be noted that a small nozzle diameter offers good precision and great resolution, though increases the printing time, which might affect the printing productivity [6]. It was suggested by Liu 2017 that a good balance should be made between the printing productivity and the printing precision. Along with this, the extrusion rate and the nozzle’s moving speed are significant during printing [11]. The nozzle movement rate can be determined by the following equation:

(1) $$v_{n}=\frac{4Q}{\pi D_{N}^{2}}$$

where $v_{n}$ is the optimal nozzle speed (mm/s), Q is the material flow rate (cm^3^/s) and D_N_ the nozzle diameter. It was indicated that a nozzle speed less than $v_{n}$ would lead to a bigger diameter material droplet than that of the nozzle, whereas a nozzle speed higher than $v_{n}$ would develop a smaller diameter material droplet than that of the nozzle. None of the above-mentioned examples are desired in the printing process. Fish surimi sculptures and chocolate figures were successfully printed, the critical movement rate of the nozzle being calculated according to this equation [11]. A few examples of food matrices and their printing parameters are centralized in Table 2.

Post-processing factors which might influence the final product’s firmness are the quantity of additives contained by the food-ink during pre-processing phase, and the possible treatments to which the products may be subjected, like baking, boiling, microwaving, or frying [11]. The food additives have the role of ensuring the stability of the product after deposition of each layer and during any post-processing phase.

The diversity in material choices and the simplicity of the device are considered the main advantages of this technique, however, the major challenges are to hold the 3D structure after printing and the difficulty of developing complex food designs [11].

### 2.2. Binder Jetting

Binder jetting was named after the adhesive liquid (glue) that holds the powder particles together during the printing process. The method has two main steps, which are repeated, using a map from a digital design file, until the object is completed. In the first step, the molding material (the food powder) is applied by the re-coater, layer by layer. The re-coater applies the powdered material on the building area with high accuracy, placing the grains exactly above each other. In the second step, the print-head applies the liquid binder, and it releases the binder material to connect with each grain of the molding. To increase the mechanical properties, the surface is usually heated by radiation, allowing the deposition of the next layer [9]. These steps are repeated, until the desired object is built up, and after the printing process is completed, the molding material is removed, and the printed object can be extracted [1]. Thereby, through this advanced concept it is easy to achieve unique and complex products in a shorter time compared to conventional methods [2].

The technology of binder jetting is used only for powder-based materials and it can be applied by the ChefJet 3D printer. Flavored and colored liquids can be customized to bind powdered material, such as sugar. Other materials that can be used are starch, powder milk, powder chocolate, etc. [9]. The printing precision is influenced by a few factors, like material properties (particle size, binder’s viscosity, and flowability), processing factors (nozzle diameter, printing rate, head types, and layer thickness) and post-processing factors (baking, heating, and removal of the surplus) [1,9]. A great advantage of binder jetting is the proficiency of printing complex 3D food structures with full-color, and the possibility of varying flavors. However, because of the few alternatives in applicable materials appropriate to this method, it can offer less nutritious products [11].

### 2.3. Selective Laser Sintering (SLS)/Hot-Air Sintering (HAS)

These technologies are centered on a laser beam or hot-air beam that is applied on a powder-based material to develop 3D objects in a short time [2]. In SLS the 3D model is well-defined by the software which convey the infrared laser to a scanner, reflecting a laser beam on the powder bed material. In that way a solid structure is made through melting the powder particles and welding them together, more exactly “sintering” [2]. The laser beam and hot-air beam, respectively, act like a heat source selectively melding (fusing) the powdered material by scanning in a cross-section motion defined by the 3D digital description encoded in the software. After the first layer of the cross-section is scanned, the powder-bed is easily lowered by one layer of thickness—usually less than 0.1 mm—and a new powder deposit is applied over the first layer. This process is repeated until the 3D object is completely formed, in the desired shape [1] and the remained powder can be re-used [47].

Usually, the printing materials are based on different mixtures of powdered components, like native wheat starch + maltodextrin + palm oil powder or sugar to obtain any kind of complex structures [3,11,26]. Fresh ingredients are not appropriate for this technology, because of the specifications of the process/printer, the only way they can be used in this technology is by dehydrating and integrating them in a powdered form [1].

The factors that influence the printing precision and the printing process are: material properties (particle size, melting temperature, and flowability), processing parameters (laser types, laser energy density, scanning speed, laser spot diameter, and layer thickness) and post-processing parameters (removal of excess powder through scraping) [1,11].

The difference between HAS and SLS consists in the power source for sintering the powder material, as it is specified in the name of each method [1,47]. Even though, these methods are limited to powder-based materials, their advantage is that any type of powdered materials can be printable. SLS/HAS are faster than the other 3DP technologies because the laser beam/hot-air beam acts straight on the powder material, without any movement of the printer bed, and complex structures can be obtained, adding the varying textures of the printed objects [2,11]. Moreover, there is no need of a post-printing hardening or use of a limited support structure, since the unsintered powder provides the needed support [26].

### 2.4. Inkjet Printing

This method is used for food decoration purposes or surface filling most frequently. The small food ink droplets are generated and placed onto the surface of the foodstuff, usually a cake, cookie, or candy, to form an image from a digital file. Inkjet printing is being described as a non-contact method, since the print-head does not touch the food during the printing process, in this way the food is protected from contamination while image filling. The appropriate materials for this technique are the ones with low viscosity, like pizza sauce or water-based inks [3].

It is necessary to manage and control a few parameters that are important in the inkjet printing process, like the material properties which needs to be supervised before printing, the compatibility of the food ink with the filling/printing surface, the rheological properties of the edible ink and the surface properties. Furthermore, the processing factors affecting the printing precision are printing rate, nozzle diameter, printing height, and printing temperature. In this method, there are no post-processing steps. As an advantage of this method, the ink droplets may comprise a wide range of colors, offering the possibility of designing unique and personalized food images and, at the same time, fast fabrication. With all, this method is appropriate only for surface design or image filling [3].

## 3. Sensory Properties of 3D-Printed Food Products

### 3.1. Texture and Structure

Food texture is the most critical factor that determines the quality of a 3D-printed food product and its acceptance [12,48,49]. Similarly, it influences the bioavailability of nutrients and functional compounds [12]. Bioavailability can be described as the fraction of a nutrient that is absorbed and available for utilization in normal physiological functions or for depositing [50].

During a meal, the food texture is perceived by the human organism through a complex system of interconnected stimuli, such as vision, hearing, touch, and kinesthetics [12]. Under these considerations and with the purpose to create and deliver highly accepted food products, that can fulfill nutritional necessities, the mechanisms involved in texture perception have been studied [48,49,51]. According to recent studies, 3DP foods can have suitable texture, a greater nutritional profile, and they appear to be aesthetically improved and more pleasant due to the extrusion method [5,49].

**Figure 2:**
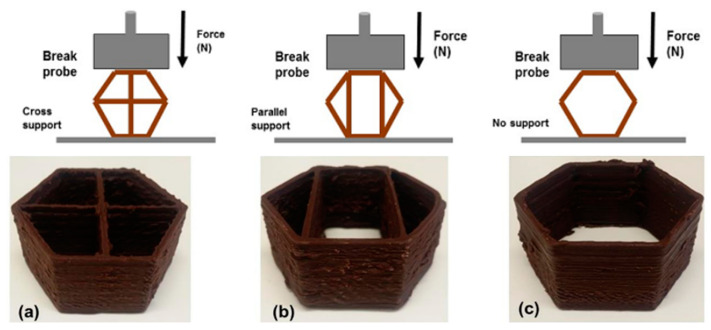
Different support structures of 3DP dark chocolate objects. Source: Three model designs of printed 3D chocolates; (**a**) hexagonal shape with cross-support; (**b**) hexagonal shape with parallel support; and (**c**) hexagonal shape with no support (Mantihal et al., 2017).

Current literature shows that texture perception of food depends also on the 3D structure [12,21,48] which may be described by information that reaches micro- and macro-scale [36]. In 3DP, the texture can be customized by controlling the internal structure of the design. A key factor in 3DFP is the internal structure of the printed object, as it needs to provide enough support structure for the product, along with holding complexity in the design. The support structure can be controlled by changing infill pattern, defined as the shape of the structure (e.g., star, line, honeycomb, etc.), as well as the percentage in the 3D printer file (G-code generator). The infill percentage (level) can vary between 1% and 100%, and it is defined as the intensity of the internal structure. Overall, the textural and mechanical properties of the 3D-printed object can be modified by the infill pattern and the infill level. Mantihal et al. (2017) used dark chocolate as raw material and developed three hexagon shapes (cross support, parallel support, and no support), as shown in Figure 2. Measuring the force required to break them, it could be noted that the structures produced by cross support were more stable than those made by parallel support [52]. Feng et al. (2020) adjusted the infill level and the infill pattern of 3D objects, assessing the effect on the texture of corn-based food formula or a mixture of yam powder and potato by-products. The results showed a linear and positive relationship concerning infill percentage and the hardness of the products after air-frying process; also, Huang et al. (2019) reported that 3D food structure printed with constant infill level but with different infill pattern presented important differences in hardness [53]. These findings show that the internal structure has a significant role in the 3D-printed product’s firmness and integrity, and this perspective is an encouraging domain for technology to develop great infill structure that can change and improve the textural properties of foodstuffs.

Furthermore, any traditional dish can be transformed into an innovative food product, due to the evolution of molecular gastronomy. This new branch of food science focuses on the physical and chemical processes that appear during cooking. Through these processes the taste of the traditional dish can be kept, customizing the shape, texture, or temperature of the dish, in order to deliver it with a completely remarkable appearance. For instance, the reinterpreted Caprese salad goes from a flat structure to a concentrated 3D dimension, surprising the visual sense. Basically, the molecular cuisine changes the food structure, with the help of ingredients like liquid nitrogen or other substances, and devices from scientific laboratories, while the taste remains unchanged [54]. It was shown that motorized extrusion (3DP method) can be combined with reverse spherification, a technique that is widely used in molecular gastronomy to obtain stable objects by gelation of fruit-based formulations, fusing molecular gastronomy with 3DFP [55].

### 3.2. Rheological Properties

Since the 3D-printing process is a matter of flow, an important element to investigate is represented by the rheological properties of the materials utilized to obtain the designed object [21]. Rheology is the science concerning the flow and deformation of materials, both solids and liquids, under the influence of stresses. The rheological descriptions used to characterize materials and help select the most suitable ones for 3D printing are steady-shear flow, oscillatory data, and temperature sweeps, creep, and recovery curves [4].

3DFP can be based on three rheological parameters: storage modulus (G’), the loss modulus (G”), and yield stress (τy), all three parameters being related to the extent of the deformation which depends on the food–ink rigidity. The storage modulus (G’) characterizes the elastic response of the food–ink and is a measure of how much energy must be put into the food–ink in order to be deformed [56]. The loss modulus (G”) is related to the material’s ability of dissipating stress through heat, and yield stress (τy) is commonly used to analyze the extrudability of food inks, and it is related to the mechanical strength of the food inks, as a higher extrusion force links to a higher mechanical strength of the food ink [21].

It is generally accepted that in 3DP the printable formulations must be shear thinning, yield stress (τy) soft material, which presents solid-like behavior [57]. The values of storage modulus (G’) must be high enough to maintain the shape, in order to hold its own weight and retain the layers deposited on top. It is also important that yield stress (τy) have high enough values to uphold printing resolution as each layer is deposited and to facilitate an easy flow during the printing process, in the same time [57]. These rheological properties depend strongly on temperature. Álvarez-Castillo et al. (2021) conducted a study about how the addition of glycerol as plasticizer influences the rheological properties of protein-based doughs (porcine plasma protein) in terms of printability [4]. A replacement of porcine plasma protein was made as well with pea protein concentrate and soy protein isolate to determine the maximum amount of porcine plasma protein that could be replaced and still obtain printable doughs. The maximum addition of pea protein concentrate was 10% and 15% of soy protein isolate. It was shown that doughs containing two biopolymers came across a noticeable increase in viscoelastic moduli and viscosity, compared to those containing only porcine plasma protein. Zhu et al. investigated the correlation between the printing behavior and rheological properties during extrusion-based 3D printing at room temperature. It was found that flow stress is a good indicator for printing stability in the investigated model systems [33]. Macronutrients behavior during extrusion of diverse food matrices was studied by Perez et al. [39], centralizing studies related to printed food materials that are rich in protein, lipid, and/or carbohydrates, and the way these nutrients impact the printability of 3DP food products. By calculating parameters, like shear modulus, the printability of multicomponent food matrix can be quantified, as well as the shape deformation of a 3DP food product can be anticipated. Moreover, the ability of a printable material to maintain its shape and obtain good resolution after printing, can be predicted by the combination of yield stress with storage modulus. A good resolution of the 3DP foodstuffs is also connected to the printing parameters, like nozzle diameter, nozzle moving speed, and extrusion rate [39]. A couple of values for these parameters are indicated in Table 2, present in the Section 2.1.

## 4. The Role of 3D-Printed Food Constituents

The most important constituents of 3DP food are binding, coloring, and fortifying agents. Each of these categories plays an important role in the process of obtaining qualitative 3DP food products. Therefore, binding agents assure the structure of the design, coloring agents play a significant role in the general acceptability of the final product, and fortifying agents confer appropriate nutritional values to the printed foodstuffs. All of the above mentioned are summarized in Table 3 and discussed in the following subchapters.

### 4.1. Binding Agents

#### 4.1.1. Xanthan Gum

Xanthan gum (C_35_H_49_O_29_) is an extracellular polysaccharide, which is produced through aerobic fermentation by various strains of *Xanthomomanas bacterium,* e.g., *Xanthomonas pelargonii* and *Xanthomonas campestris*. It was approved by the United States Food and Drug Administration (FDA) as a food additive, without any restrictions of use in the food industry, and it was registered as an emulsifier and a stabilizer in the Code of Federal Regulations (CFR) [65,66,67].

Xanthan gum is mostly utilized in the food industry as a stabilizer, an emulsifier, and a thickener, due to its outstanding characteristics to obtain high viscosity even at a low concentration, good stability under acidic and alkaline conditions, and excellent solubility in hot/cold water. Therefore, the production of xanthan gum from *X. campestris* grew with an annual rate of 5–10%, and it is expected to reach 30,000 tons per year [65]. This natural biopolymer is a cream-colored, odorless substance that can be used in 3DFP due to its remarkable properties like biocompatibility, great pseudoplastic properties, thermostability, and as an immunological agent. Its applicability is found in many other domains as well, such as medicine, biomedical engineering, and waste-water treatment [65]. A recent study investigated the 3D printability of gels based on xanthan/konjac gums, regarding the effect of composition on rheological and textural properties. Higher values of G′, G″, and η* were exposed/presented for the formulations with greater content of xanthan gum and glucomannan, and lower syrup concentration [6]. Furthermore, it was validated that the addition of xanthan gum in k-carrageenan-based inks increased the gelation temperature, viscosity, yield stress, and G′, it reduced time-dependence of modulus, and improved the shear-thinning behavior [66].

#### 4.1.2. Pectin

Pectin (C_6_H_10_O_7_) is an anionic, water-soluble biopolymer and one of the main structural acidic hetero-polysaccharide of terrestrial plant cells. It can be extracted from many food industrial processing by-products, such as fruits and vegetable pomaces, as it is one of the major constituents of citrus fruits, apple, and mango. A rich source of pectin is sugar beet pulp residues resulting from sugar extraction [68].

As it was reported by the Food and Agricultural Organization of the United Nations and World Health Organization experts on food additives that there is no limited daily intake that has been ascertained for pectin, since it is considered safe and non-toxic. Pectin is used in the food and beverage industry as a thickening and gelling agent, texturizer, emulsifier, colloidal stabilizer, and, recently, for applying coatings on fresh and cut fruits and vegetables. Under appropriate conditions, pectin can form gels alongside sugar and acid, due to its configuration of water-soluble pectinic acids with variable methyl ester content [68].

Vancauwenberghe et al. (2017) have presented that by using different pectin-based food-inks/formulations, various food objects could be printable, with adjustable microstructure and textural properties. It was shown that the pectin concentration was the main factor which determined the firmness and strength of the printed object. Moreover, together with sugar, it increased the viscosity and influenced the build quality [58].

#### 4.1.3. Chitosan

Chitosan (C_56_H_103_N_9_O_39_) is a poly-cationic, biocompatible, and biodegradable biopolymer, approved by the European Food Safety Authority (EFSA) as safe for consumption. It is obtained by the alkaline hydrolysis of chitin, which is the main structural component of the exterior skeletons of shellfish, and it can also be found in other species, for example, insects and fungi [68,69]. The most extracted form of chitosan is α–chitosan obtained from shrimp shell and crab shell wastes chitin, where it is found in the proportion of 70%. Its major bioactive property is related to antimicrobial activity confirmed by numerous studies (Kanatt et al., 2012; Pranoto et al., 2005; and Tripathi et al., 2009). Its muco-adhesive character has led to the creation of biodegradable labels, and it gained the attention of researchers in the 3DP industry, such as a 3D-printed chitosan scaffold that was developed [68,70,71]. However, chitosan is pH-dependent regarding solubility, a fact that could limit its use in alkaline food products. The chitosan/halloysite nanotubes/tea polyphenol (CS/HNTs/TP) nanocomposite films were developed through 3DP technology, and they presented good antioxidant and antibacterial properties. This new approach provides a promising method of obtaining natural, antioxidant, and antibacterial food packaging [59].

### 4.2. Coloring Agents

Color is considered one of the essential characteristics of the sensory quality of a food product. It influences the perception of the consumer on their judgement of the product and its other attributes, such as flavor [72,73]. The food industry uses allowed food colorants to improve the appearance of their products. Therefore, the food-colorant industry is estimated to reach USD 512 million by 2023, with the annual growth rate of 5.7% [62]. Moreover, there are a few safety concerns related to synthetic food colorants, which force the industry to replace them with natural colorants [62]. In addition, natural food colorants can contribute to assuring functional properties in foodstuffs. Although, detailed studies need to be conducted linked to the entire industrial process involving the natural dyes, to guarantee color maintenance [16,74].

#### 4.2.1. Anthocyanins

Anthocyanins (E-163) are special nutrients composed of an aglycone anthocyanidin and sugar moieties belonging to the flavonoids class [16,60]. They are water-soluble pigments, mainly found in flowers and fruits of plants (e.g., raspberries, eggplants, blackberries, and rose petals) producing different shades of red, purple, and blue in different plant organs [75]. Alongside the property of coloring agents, anthocyanins show significant antioxidant, anti-inflammatory, and lipid-regulation functions and are able to improve the stress resistance of plants [76,77].

**Figure 3:**
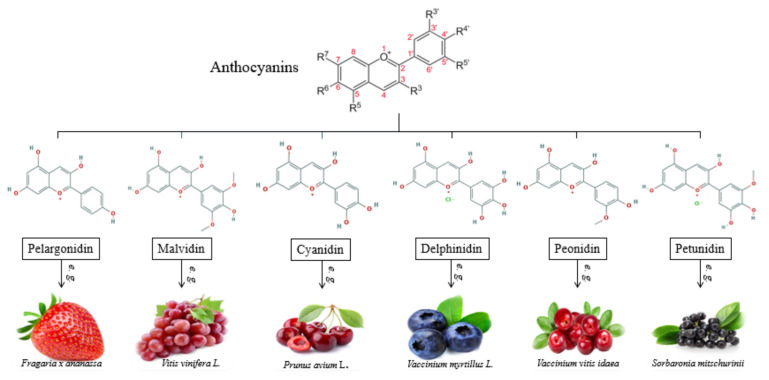
Chemical structures and sources of natural anthocyanins used in the food sector.

Furthermore, anthocyanins are safe for consumption as natural pigments, presenting great pliability in food industry as potential replacers of synthetic colorants [75]. According to [76], more than 20 types of anthocyanidins have been identified, out of which, six are prevalently used in the food sector (cyanidin, peonidin, pelargonidin, petunidin, malvidin, and delphinidin), as presented in Figure 3. A valuable source of anthocyanins are eggplant by-products, since it is approximated that over 10 million tons of this kind of by-products is generated yearly. The peel of eggplants is rich in delphinidin-derived anthocyanins with great antioxidant, antimicrobial, and anticancer properties, also playing an important role in human nutrition [75].

Recently, edible rose petals, with high anthocyanins content, were combined with sodium alginate to develop a new type of snack food by using the 3DP technology. Beautiful 3D figures were developed with wonderful colors of purple, depending on the added concentration of rose petals to the formulations [60]. This study underpins the multiple possibilities of using vegetal-derived colorants in 3D-printed food products and encourages future directions in the applicability of recovered colorants/nutrients from by-products of the agro-industry.

#### 4.2.2. Chlorophyll

Chlorophylls (E-140) are one of the most obvious natural pigments on Earth. They are extensively distributed in plants, algae, and bacteria, having an important role in photosynthesis [16,78]. They are a great basis of antioxidants, like vitamins A, C, and E, which can neutralize free radicals in the body. Even though these pigments are vulnerable to light and oxidation, they possess color-changing, antioxidant, and antibacterial properties [79].

The use of copper chlorophylls (E-141i) as food additives, is authorized by the European Union (Regulation EC 1333/2008), and by other countries, such as Japan and China. They are produced by the main food-coloring companies and are used in different food products for natural green coloration, like ice-creams, dairy products, cookies, jellies, and others [62].

A cereal snack containing different microalgae (*Spirulina* spp. and *Chlorella* spp.) concentrations, was developed and it was printed through 3DP technology. The study reflected the effect of microalgae biomass on rheology, texture, and color of the final product. The results showed varied color of the printed snacks, depending on the concentration level of microalgae added to the formulations. The pre-established form of the cereal snack suffered color changes after being subjected to baking, the post-processing phase, due to the pigment degradation of chlorophyll, since it is thermolabile [61].

#### 4.2.3. Spirulina

Spirulina (*Athrospira*) is a blue–green algae, belonging to *Cyanobacterium* species, which is cultivated for its nutritious content in proteins, antioxidants, phytonutrients, probiotics, and nutraceuticals [16,80]. Due to its composition, spirulina is easily absorbed by the human body and has great health benefits, reducing oxidative stress, and preventing diabetes, cardiovascular diseases, and other non-communicable diseases [80]. It is used in medicine, cosmetics, and waste-water treatments, as well. The two main species of spirulina are *Athrospira platensis* and *Athrospira maxima* [80]. *A. platensis* is rich in phycocyanin, which is a blue protein, accepted by the US FDA as a natural dye and usable in sweets. Also, in the European Union, phycocyanin (spirulina extract) is considered a non-toxic food colorant. However, the industrial application of phycocyanin might be limited due to the deficit of stability to light, high temperatures, and pH variations, plus high-cost production [16].

The spirulina extract was introduced in a cookie dough appropriate for extrusion in order to develop enriched cookies with innovative appearance [80]. The encapsulated form of the extract was showed to improve antioxidant activity and color maintenance during storage when compared to the other formulations, suggesting its applicability in 3DFP.

### 4.3. Fortifying Constituents

**Figure 4:**
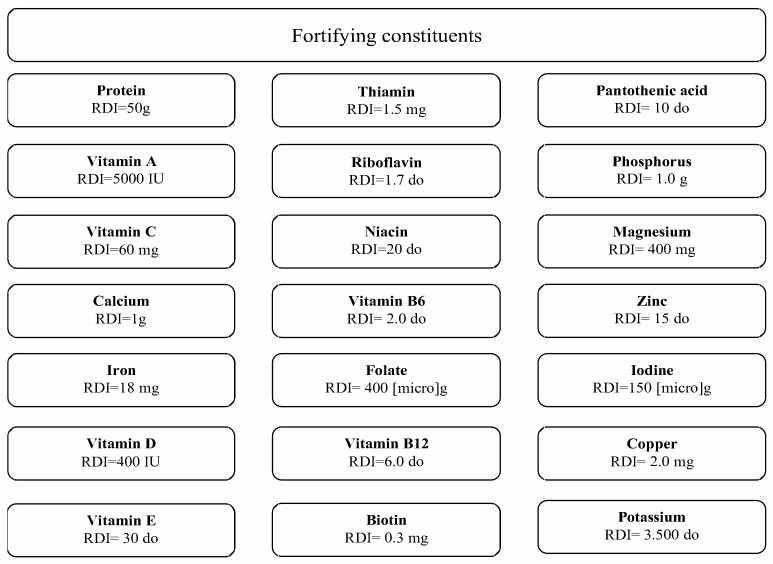
Nutrient list indicated by the U.S. Food and Drug Administration that may be added to functional food products. Legend: RDI = Reference Daily Intake for adults and children of 4 or more years of age; g = gram; IU = international unit; mg = milligram; and [micro]g = microgram. Source: https://www.accessdata.fda.gov/scripts/cdrh/cfdocs/cfcfr/CFSearch.cfm?fr=104.20 accessed on 5 July 2021.

The concept of “fortifying” refers to functional foods containing ingredients that offer health benefits and extend the final product’s nutritional value. Some food products contain supplements or other additional ingredients designed to improve health, like vitamins, minerals, probiotics, fiber, and others, as is presented in Figure 4. Nutrient-rich ingredients like fruits, vegetables, nuts, seeds, and grains are often considered functional foods as well [81,82]. Food fortifiers like macro- and micro-nutrients can be added from agri-food production chains or obtained from by-products [81,83,84].

With the help of 3DFP technology, these fortified foods are easily developed, being characterized by the right nutritional intake for each individual’s need. Oliveira et al. (2021) investigated the antioxidant activity of 3DP cookies enriched with encapsulated polyphenols. They showed that the bioactivity and total phenolic content was improved by 115% and 173%, respectively, for the four-layer cookies with 30% infill, comparing to the extract-free cookies [63,64] incorporated cricket and pea protein powder as additives in 3DP mashed potatoes formulations, showing how they can influence the printability and the nutritional content of the mashed potatoes samples. The results show that the samples were enriched with significant amounts of protein, fat, fiber, vitamins, and minerals and the highest shape fidelity prints were for a water to additive ratios of 2 and 3, respectively [64].

#### 4.3.1. Personalized 3D-Printed Food

3DFP can be utilized to develop a personalized nutrition for people with special dietary necessities. This means that the selected ingredients, introduced in the 3D printer to compose the meal, have a specific contribution, and are tailored for the individual’s nutritional needs. For example, a person with chronic kidney disease needs a meal with low potassium content, therefore 3DP offers the possibility of integrating only this kind of ingredients into the meal, and have it delivered in a personalized form [5]. Similarly, this technology can provide diverse food designs for people with chewing and swallowing struggles, giving them a different option for the “ice-cream-scooped” pureed food.

“Sarcopenia” and “dysphagia” are the most common diseases that occur among elderly people and are related to chewing and swallowing difficulties. Sarcopenia, which is Greek for “poverty of flesh”, is characterized by progressive and generalized loss of skeletal muscle mass causing a reduction of physical strength that can lead to functional impairment, poor quality of life, extension of hospitalizations, and even death [8,85]. It is estimated that by 2030 the worldwide population of people aged 60 years and over will reach 1400 million, and 2100 million by 2050, this number includes the 202 million people aged 80 years and over by 2030, and 434 million by 2050 [8]. The focus of research is on therapeutic strategies that might be efficient against this age-linked disease. Luo et al. (2017) highlighted that one strategy might be associated with dietary interventions like supplying the affected population with essential specific nutrients, such as proteins, fibers, vitamins, etc. This is where 3DFP can prove beneficial by suggesting innovative controlled-composition foodstuffs with adjusted textures and flavors [85]. The printed food may fulfill requirements better according to the texture-modification guidelines of the International Dysphagia Diet Standardization Initiative Framework (International Dysphagia Diet Standardization Initiative, 2019).

Dysphagia means “difficulty in swallowing”, and it affects 15–25% of aging society. The prevalence of this disease is higher amog patients who had Parkinson’s disease, stroke, paralysis, etc. Therefore, one of the most significant applications of 3DFP depends on the design of customized meals aimed for elderly patients dealing with these difficulties [86], since the food given to these patients has to be texture-suitable with improved appearance, such as purees or thickened fluids [8,87].

The alternative would be to receive a meal with modified texture, proper for their specific needs, yet with an appealing appearance that looks like the original product. Dick et al. (2019) developed three hypothetical meat models, such as steak, beef patty, and sausage, and showed that recombined meats (steaks) can be 3D printed from soft meat paste, lipids, and alternative ingredients to come close to the original flavors and nutrients of a beefsteak [86,88].

Furthermore, these new technologies could be valuable to improve food security and overcome global famine. Worldwide, there still are a number of countries affected by famine. Some of them continue to have extreme cases of starvation, and the exposed population has specific needs. Through 3DFP, maximized nutritional food products could be developed from different sources of nourishment, such as meats, lupine seeds, insects, and algae [1,8,89]. These products could be manufactured to be visually appealing, with an enriched nutritional profile and in different forms and colors [89].

When it comes to insects, they are considered a source of good quality proteins, often referred to as “the novel protein” by scientific researchers. Besides proteins, they are also rich in lipids, vitamins, and minerals [36]. However, many food allergies are related to proteins, thus, EFSA evaluates the allergenicity level, in order to decide if insect-based proteins should be authorized for the European dinner plate. These allergic reactions can be caused by an individual’s sensitivity to insect-based protein, cross-reactivity with other allergens, or residual allergens from insect feed (e.g., gluten). Furthermore, the EFSA experts are concerned about the consumers’ acceptance of foodstuffs with incorporated insects, due to the repellent thought of eating insects (the so-called “yuck factor”). Although, with time and exposure these kinds of attitudes can be improved. In order to support this trend, the addition of a small amount of insects in different forms, like dried powder or pastes, etc., into 3DP food products, might be helpful [36].

Additionally, children and teenagers are part of another category with special dietary requirements, being deficient in nutrients such as protein, vitamins, and minerals (especially iron and calcium) [87]. Therefore, since 3DFP is applicable for acquiring attractive food products with improved nutritional profile, this can be a possible solution for creating healthy snacks with unique shapes and, at the same time, reaching the nutritional requirements for each individual [20,87]. 3DP foodstuffs rich in macronutrients are presented in Table 4.

##### Future Perspectives

EFSA summarized the Novel Food Regulation in 2012, including herbal products derived from plants, algae-based food, non-indigenous fruits, and a variety of edible insects. These novel foodstuffs need to be evaluated in terms of nutritional profile, from a toxicological, microbiologic, chemical point of view, etc., with the intention of the replacement of traditional sources of animal proteins. According to EFSA, there could be significant environmental and economic advantages by introducing alternative sources of protein, that require less feed, produce less waste, and result in fewer greenhouse gas emissions.

Bioactive compounds found in food processing by-products could be integrated into innovative, functional nourishment, by combining molecular gastronomy and 3DFP, enhancing the inner biological activities, nutritional profile, and the appearance of the food, and addressing sustainable approach to food-waste management at the same time. Recently, a comprehensive review regarding the viscoelastic materials and composites used in food manufacturing concluded that there is high potential to connect the best of optimized 3D printing in food manufacturing using principles of additive layering, that emphasize with the human need for gastronomic and nutritional satisfaction [95].

## 5. Conclusions

3DFP is an expanding area of food processing and gastronomy with very diverse possibilities to branch out much further, from personalized nutritional care food products to tailored food products to deal with specific ailments. The critical factors influencing consumer acceptance over novel food formulations is related to the food aspect (color, shape), sensory properties (flavor, texture), nutritional composition (macro- and microelements), and costs. Currently, most scientific research focuses on structure and composition, being in an incipient stage of developing the concept of 3DFP. Thus, for a general acceptability of the concept, it would be desirable to analyze consumer preference related to the new ingredients and their functionality. Simultaneously with the sensory analysis (e.g., hedonic tests) of the 3DP food products, the consumer has to be informed about the whole printing process and the beneficial effects of functional foods on health.

Any type of functional ingredients can be integrated in new food products, as long as an appropriate powdered form of the matrix is developed (mash size of the particles, dried powdered), and the suitable 3DFP technology is selected (e.g., extrusion, SLS, HAS, or binder jetting). Furthermore, regarding the circular economy action plan of the European Union, bioactive compounds recovered from diverse agro-industrial by-products can be incorporated, like pectin from apple wastes, chitosan from crustacean processing, and carotenoids from tomato agro-industrial by-products. At the same time, these ingredients can add value to the new food products through their biological activities, like antioxidant properties, antimicrobial and anti-inflammatory functions, fulfilling the purpose of sustainable bio-economy.

## Figures and Tables

**Table 1:** Recently developed food products through 3DP technologies.

Food Products	Printing Method	Printer	Objectives/ Findings of the Study	Reference
3D-printed cereal-snack bar	Extrusion	3D printer Delta 2040 equipped with a clay extruder kit 2.0	- Cereal-snacks with different texture properties (porosity fraction, hardness) by means of 3D-printing technology.	[12]
3D-printed buckwheat dough with yellow flesh peach, enriched with complex coacervates microcapsules	Extrusion	SHINNOVE-D1 3D food printer	- Feasibility of using microwave heating as a stimulus and microcapsules (gelatin-gum Arabic-oil complex coacervates) as a stimulus–response material to realize color and aroma changes in 3D-printed buckwheat dough containing yellow flesh peach.	[18]
Button mushroom	Extrusion	3D printer CARK	- Development of fiber-enriched snacks from mushrooms, highlighting the use of sustainable alternative food sources for the preparation of healthy, customized snacks.	[19]
Cookies	Extrusion	A FoodBot 3D-printer	- Investigation of different printing and product parameters (structure, microstructure and hardness) on the rheological properties of 3D-printed cookie dough.	[20]
Cookie dough	Extrusion	Modified 3D printer by replacement of the nozzle with a digital air syringe dispenser.	- Effects of flour and fat types on the printability and post-processing capacity of cookie dough to establish an appropriate methodology for structurally stable complex 3D.	[21]
Cookies with microalgae	Extrusion	3D food printer equipped with a pasta extruder nozzle designed for food materials	- Coloristic, rheological, and textural characteristics of cookie doughs by the addition of two microalgae (*Arthrospira platensis* and *Chlorella vulgaris*) in 3D-printed cookies.	[17]
Dough	Extrusion	Extrusion system with an air pump and an X-Y-Z position device.	- The effect of material composition on the quality of 3D-printed food using wheat flour, freeze-dried mango powder, olive oil, and water.	[22]
Emulsion with whey protein isolate and soy oil	Extrusion	3D food printer	- The effects of oil fraction on the visual, rheological, and microstructural properties of Pickering emulsions were investigated.	[23]
Gels based on xanthan/ konjac gums	Extrusion	Commercial 3D printer equipped with a paste extruder nozzle to work with food ingredients	- The printability of gels based on xanthan/konjac gums when affected by printing variables (printing temperature, rheological, and textural properties), analysing the composition of the product.	[6]
Grinding and milling fractions of rice husk with the addition of guar gum	Extrusion	3D food printer CARK	- Conversion of non-printable rice husk to printable form by the addition of guar gum, which can be further utilized in food packaging, reducing the dependency on non-degradable petroleum-based plastics.	[7]
Mashed potatoes with probiotics (Bifidobacterium animalis subsp. Lactis)	Extrusion	Two-nozzle printer	- Investigation of printing parameters and storage time on the viability of probiotics in 3D-printed mashed potatoes.	[24]
Mixture of whey protein isolate and gellan gum (GG)	Extrusion	Focus 3D food printer (byFlow)	- Establishment of a set of tools and procedures allowing an objective evaluation and prediction of the printability of edible biopolymer blends. The tools are applied to phase-separated inks to elucidate intrinsic properties required to improve the printability of whey protein isolate.	[25]
Mixture of 50% native wheat starch + 40% maltodextrin + 10% palm oil powder	Selective laser sintering	-	- 3D-printed samples were mechanically characterized by means of compression testing. The observed phenomena are captured in a constitutive model that describes the large deformation behavior and the brittle failure of the material.	[26]
Potato puree	Extrusion	Commercial 3D printer equipped with a paste extruder nozzle to work with food ingredients	- Analysis of the rheological and textural properties on the printability of potato puree affected by printing variables (printing temperature and composition of the potato puree).	[27]
Potato starch	Extrusion —hot	SHINNOVE-S2 printer	- Investigation of the changes in structure and rheological properties of potato-starch paste during hot-extrusion 3D printing correlated with different concentrations and printing temperatures.	[28]
Powdered milk	Extrusion —cold	Pneumatic Direct Ink writing (DIW) printer	- The printability of a milk ink and other edible inks, without incorporated additives, at room temperature.	[29]
Rice starch	Extrusion	3D printer CARK	- Effect of nozzle size, print, and motor speed on the printability of rice starch, considering uniformity and ease of extrusion. Thread quality, binding property, finishing, texture, layer definition, shape, dimensional stability, and appearance were observed as well.	[30]
Snack bars (Acceptance study)			- The acceptance of 3D-printed food acceptance in a real-life military setting. Over a period of four weeks, soldiers consumed and evaluated multiple recovery snack bars.	[31]
Soy protein isolate, pumpkin, and beetroot mixture	Extrusion	3D-printing system	- Examination of the stimulation at different pH to alter color, texture, and flavor of soy protein isolate, pumpkin, and beetroot mixture as 3D-printed food product.	[32]
Tomato paste	Extrusion	ByFlow 3D printer	- Potential correlations between printability of formulations and simple rheological properties. The tomato paste was used as a model system.	[33]
High-oil-content (up to 37%) pastes	Extrusion	ByFlow 3D printer	- The effect of oil content on the printability of a model food paste.	[34]
Fresh and frozen vegetables with addition of hydrocolloids	Extrusion	FOODINI	- Categorization of different vegetables, having dissimilar water and starch content, to render them 3D printable and designing visually pleasing foodstuffs for dysphagic patients.	[35]

**Table 2:** Printing parameters of different foodstuffs.

Type of Product	Printing Parameters							Reference
	Nozzle Diameter	Nozzle Height	Nozzle Moving Speed	Extrusion Rate	Printing Pressure	Temperature	Time	
Surimi gel	2 mm	5 mm	28 mm/s	0.003 cm^3^/s	NS	25 °C	NS	[45]
Plasma protein-based doughs	1.5 mm	NS	NS	0.0024 mL/s	NS	20 °C	6.5 min	[4]
Buckwheat dough with yellow flesh peach	1.2 mm	NS	20 mm/s	35 mm^3^/s	NS	25 °C	NS	[18]
Vegemite and Marmite	NS	NS	NS	NS	15 psi (103 kPa)	25 °C	NS	[43]
Yam-Potato by-product paste	1.2 mm	NS	20 mm/s	22 mm^3^/s	NS	23 ± 1 °C	NS	[46]
15–25% potato starch	0.8 mm	1.0 mm	30 mm/s	NS	NS	70 °C	NS	[28]
Beef	N_1_ = 2 mm/N_2_ = 1 mm	NS	20 mm/s	NS	NS	23± 1 °C	6.37 min, up to 10 min	[39]

NS—not specified.

**Table 3:** Functional ingredients used in 3D-printed food products.

Function	Constituent	Source	Application	Reference
Binding agent	Xanthan gum	*Xanthomonas* *campestris*	Printable gels based on xanthan gum.	[6]
	Pectin	Fruits and vegetable pomaces (e.g., apple, sugar beet pulp, etc.)	3D-printed objects from pectin-based food-ink.	[58]
	Chitosan	Shellfish, fungi, insects	3D-printed chitosan/halloysite nanotubes/tea polyphenol nanocomposite films.	[59]
Coloring agent	Anthocyanins	Flowers and fruits of plants (e.g., raspberry, eggplant, etc.)	3D-printed snack food with the addition of rose petals.	[60]
	Chlorophyll	Plants, algae and bacteria	3D-printed cereal snack with the addition of *Chlorella*.	[61]
	Spirulina	Blue-green algae	Cookie dough with spirulina extract.	[62]
Fortifying constituents	Bioactive compounds (polyphenols, antioxidants, and essential oils)	Plants	Cookies enriched with encapsulated polyphenols.	[63]
	Protein	Meat, eggs, dairy products, etc.	Incorporated cricket and pea protein powder in 3DP mashed potatoes formulations.	[64]

**Table 4:** 3DP foods rich in macronutrients.

Nutrients	Food Materials	Reference
Protein	Meat	[90]
	Cricket protein	[91]
	Pea protein	[92]
Lipid	Chocolate	[52]
	Cheese	[93]
Carbohyrates	Potato puree	[27]
	Dough	[22]
	Cookies	[94]

## Data Availability

The study did not report any data.
